# Increased peptidylarginine deiminases expression during the macrophage differentiation and participated inflammatory responses

**DOI:** 10.1186/s13075-019-1896-9

**Published:** 2019-04-30

**Authors:** Ning-Sheng Lai, Hui-Chun Yu, Chien-Hsueh Tung, Kuang-Yung Huang, Hsien-Bin Huang, Ming-Chi Lu

**Affiliations:** 1 Division of Allergy, Immunology and Rheumatology, Dalin Tzu Chi Hospital, Buddhist Tzu Chi Medical Foundation, No. 2, Minsheng Road, Dalin, 62247 Chiayi Taiwan; 2 School of Medicine, Tzu Chi University, Hualien City, Taiwan; 3 Department of Life Science and Institute of Molecular Biology, National Chung Cheng University, Minxiong, Chiayi Taiwan

**Keywords:** Citrullination, Macrophages, PAI-2, PADI2, PADI4, PSMB1

## Abstract

**Objective:**

To investigate the expression of peptidylarginine deiminases (PADIs) during macrophage differentiation and its role in inflammatory responses.

**Methods:**

The protein expression of PADI2, PADI4, and citrullinated histone 3 in U937 cells, differentiated macrophages, and macrophages stimulated with lipopolysaccharides (LPS) were analyzed by Western blotting. Three PADI inhibitors were used for assessing their effects on the secretion of proinflammatory cytokines in macrophages. The differential expressed citrullinated proteins during macrophage differentiation were probed by self-prepared anti-citrullinated protein antibodies, and the reactive bands were sent for proteomic analyses. Transfection studies were conducted to search for the functions of specific proteins. A specific protein was cloned and citrullinated for its protein binding study.

**Results:**

The expression of PADI2 and PADI4 markedly increased during macrophage differentiation, whereas the formation of citrullinated histone 3 increased after stimulated with lipopolysaccharides. Three PADI inhibitors suppressed the LPS mediated proinflammatory cytokines secretion, but did not affect the expression of PADI2 and PADI4. Plasminogen activator inhibitor-2 (PAI-2) was citrullinated during macrophage differentiation. The expression of *PAI-2* increased during macrophage differentiation and further increased after stimulated with LPS. Suppressed *PAI-2* expression decreased the expression and secretion of proinflammatory cytokines. Decreased PADI2 expression also suppressed the expression of PAI-2 and protein levels of citrullinated PAI-2. The citrullination of PAI-2 inhibited its binding ability to proteasome subunit beta type-1 (PSMB1).

**Conclusion:**

PADI2 and PADI4 protein levels increased during the macrophage differentiation resulting in protein citrullination, including PAI-2. The increased expression of *PAI-2* promoted inflammatory response, and the citrullination of PAI-2 impaired its binding to PSMB1. Therefore, protein citrullination could play a critical role in macrophage differentiation and function.

## Introduction

Rheumatoid arthritis (RA) is a chronic systemic autoimmune disease characterized by persistent inflammation of the joints that can lead to disability. Recently, the presence of anti-citrullinated protein antibodies (ACPAs), a very specific biomarker for the diagnosis of RA, was reported to participate in the pathogenesis of RA [1, 2]. The molecular targets of ACPAs are citrullinated proteins, which are catalyzed by peptidylarginine deiminases (PADIs) [3]. There are five PADIs expressed in human with PADI2 and PADI4 highly expressed in leukocytes [4]. Increased PADI activity and protein hypercitrullination were noted in patients with RA and their first-degree relatives [5, 6]. In addition, in murine collagen-induce arthritis, *PADI4* knock out was shown to reduce joint inflammation [7]. *PADI2* knock-out mice showed a reduced disease activity in murine tumor necrosis factor (TNF)-α-induced arthritis [8]. Genetic studies also showed that functional haplotypes of *PADI4* and single nucleotide polymorphism of *PADI2* gene were associated with the risk of developing RA [9, 10]. Therefore, PADI2 and PADI4 might contribute to the pathogenesis of RA.

During the process of monocyte/macrophage precursor cells differentiating to mature osteoclasts, the expression of PADIs and the subsequent formation of citrullinated proteins were increased [11, 12]. Similarly, increased protein expression of PADI2 during the differentiation of monocytes to macrophages using PBMCs from RA patients and controls were reported [13]. The expression of PADI2 and PADI4 was found to increase in human monocytes after being stimulated by activated T cells [14]. We believe that the increased PADI expression during the differentiation of macrophage could lead to citrullination of certain proteins, and resulting in inflammatory responses. Thus, we hypothesized that during monocyte differentiate to macrophage, PADIs expression would increase, and certain protein(s) participating in the inflammatory response would be citrullinated with altered protein-binding ability.

## Material and methods

### Cells cultured with different PADI inhibitors

The study was approved by the institutional review board of Buddhist Dalin Tzu Chi Hospital, Taiwan (No. B10604013). U937 cells (American Type Culture Collection, Manassas, VA, USA) was induced to differentiate into macrophage-like cells by cocultured with 500 ng/mL phorbol 12-myristate 13-acetate (PMA; Sigma-Aldrich, St. Louis, MO, USA) at 37 °C in a humidified atmosphere containing 5% CO_2_ for 48 h. The obtained cells are hereinafter referred to as “differentiated macrophages”. The differentiated macrophages were then cocultured with lipopolysaccharides (LPS; 20 ng/mL, Sigma-Aldrich, St. Louis, MO, USA) in the presence of one of the three different PAD inhibitors, including Cl-amidine (10 μM), ruthenium red (RUR; 10 μM), or sanguinarine (SANG; 10 μM) (all from Sigma-Aldrich) for 24 h at 37 °C in a humidified atmosphere containing 5% CO_2_. The culture supernatants were collected and stored at − 80 °C for enzyme-linked immunosorbent assay (ELISA). The cells were harvested for Western blot analysis.

### Detection of the expression of CD86 by flow cytometry

The positivity of CD86 was determined by stained phycoerythrin-conjugated mouse monoclonal antibody against human CD86 (Taiclone, Taipei, Taiwan) or isotype control (BD Biosciences, Franklin Lakes, NJ, USA) in cells analyzed by flow cytometry (FACScan, Becton Dickinson, Franklin Lakes, NJ, USA) using Lysis II software.

### ELISA

The concentration of cytokines in the culture supernatants was determined using an ELISA kit (BD Biosciences) according to the manufacturer’s specification.

### Preparation of cell lysates and nuclear extract

Cells were lysed with 1% NP-40 (Sigma-Aldrich) in the presence of a proteinase inhibitor cocktail (Sigma-Aldrich) and a phosphatase inhibitor cocktail (Thermo Fisher Scientific, Waltham, USA). Nuclear extract was prepared using Nuclear Extract Kit (Active Motif, Carlsbad, CA, USA) according to the manufacturer’s protocol. The protein concentration of these samples was measured using the Bradford method. The culture supernatants were collected and stored at − 80 °C for further analysis.

### Purification of ACPAs and ACPA-depleted [ACPA (−)] sera from pooled ACPA (+) RA sera

ACPAs and ACPA (−) sera were purified according to the method described previously [15]. In brief, pooled serum containing high concentration of ACPAs were purified by affinity chromatography using an ÄKTA purifier 10 (GE Healthcare) with UV detection at 280 nm for collection of desired fractions. The ACPA (−) sera was the initial effluent not bound to the CCP-affinity column. The procedure was repeated for several cycles until no CCP-binding activity (< 7 IU/mL) was detected.

### Western blot analysis

Cell lysate or cell nuclear extract were electrophoresed and transferred to a polyvinyllidene difluoride (PVDF) sheet (Sigma-Aldrich). The membranes were non-specifically blocked in 1% skim milk solution and incubated with the primary antibodies for PADI2, PADI4, PAI-2, lamin A/C, and glutathione S-transferase (GST; all from Abcam, Cambridge, UK), phospho-NF-κB p65 (Ser536) (Cell Signaling Technology, Danvers, MA, USA) followed by respective HRP-conjugated secondary antibodies (Jackson ImmunoResearch Laboratories, West Grove, PA, USA). Blots were visualized by chemiluminescence reaction (ECL; GE Healthcare, Little Chalfont, UK). Respective band intensities were measured using Image J (version 1.42; http://rsb.info.nih.gov/ij).

### Protein identification by MALDI-TOF mass spectrometry

Cell lysate of U937 cells capable of reacting with ACPAs were excised and submitted for peptide identification (Mission Biotech, Taipei, Taiwan). The gels were digested and fractionated by LC Packings Nano Flow HPLC System (Thermo Scientific Dionexin, Sunnyvale, CA, USA). The desired samples were analyzed by Microflex MALDI-TOF MS (Bruker Daltonics, Billerica, MA, USA).

### Transfection of small interfering RNAs (siRNAs)

Cells were transfected with siRNAs targeting PAI-2 or scramble oligonucleotides (Invitrogen, Carlsbad, CA, USA) using the conditions previously described [16] and then cultured with the condition described above for 24 h to perform real-time reverse transcription-polymerase chain reaction (RT-PCR) and ELISA analysis, or for 48 h to perform Western blotting.

### Measurement of cytokine expression levels by RT-PCR

Total RNA was extracted using the Quick-RNA MiniPrep kit (Zymo Research, Irvine, CA, USA) according to the manufacturer’s protocol. RNA concentration was quantified using a spectrophotometer (NanoDrop 1000, Thermo Fisher Scientific). The mRNA expression levels of *IL-1β*, *TNF-α*, and *IL-6* were quantified by real-time RT-PCR by one-step RT-PCR kit (TaKaRa, Shiga, Japan) with an ABI Prism 7500 Fast Real-Time PCR system (Applied Biosystems, Waltham, MA, USA). Relative expression levels of mRNA were defined by the following equation: (39 − threshold cycle [Ct] after adjusted by the expression of U6 small nuclear RNA [snRNA]).

### Inhibition PADI2 protein expression

U937 cells were electroporated with PADI2 short hairpin RNA (shRNA) plasmid (Santa Cruz Biotechnology, Dallas, TA, USA), which contained three 19–25 nt shRNA designed to knock down *PADI2* expression. Control shRNA plasmid (Santa Cruz Biotechnology) encoded with a scrambled shRNA sequence that would not lead to the specific degradation of any cellular message were transfected into U937 cells by Gene Pulser MXcell electroporation system (Bio-Rad Laboratories, Hercules, CA, USA) and then selected by culturing with 0.3 μg/mL puromycin (Invitrogen) as previously described [17].

### Preparation of PAI-2 and GST-tagged proteasome subunit beta type 1 (PSMB1)

Cloning of PAI-2 and GST-tagged PSMB1 was based on the methods described previously with modifications [17]. Full-length *PAI-2* and *PSMB1* cDNA was amplified by PCR. The resulting products were digested with *Xho*I and *Eco*RI (New England Biolabs, Ipswich, MA, USA) and subcloned into *E. coli* BL21 (DE3). It was then transformed with recombinant pET-28a encoding PAI-2 or pGEX-4 T1 encoding PSMB1. The transformed bacteria were grown in LB broth with ampicillin (0.1 g/L) and induced with 1 mM isopropyl-β-thiogalactoside for 4 h at 37 °C. Bacteria were harvested by centrifugation, resuspended in 100 mL of 20 mM Tris-HCl buffer (pH 7.9) containing 0.5 M NaCl, 0.2 mM phenylmethylsulfonyl fluoride, 0.02% NaN3, 4 mM benzamidine, and 0.5 mM imidazole, and lysed using a French press. PAI-2 was purified from the crude lysate by sequential separation on nickel Sepharose column. GST tagged PSMB1 was purified by GSH-Sepharose column (2.5 cm × 10 cm) using linear gradient from 250 mL of Buffer A (20 mM Tris-HCl, pH:7.5, 0.3 mM EDTA, 4 mM benzamidine, 0.3 mM DTT, 0.02% NaN_3_, 0.15 M NaCl) to 250 mL of Buffer A with 10 mM GSH. The purified protein fractions were pooled, concentrated by ultrafiltration and dialyzed against polymerization buffer (5 mM Tris-HCl [pH 7.5], 2 mM CaCl_2_, 0.1 M KCl, 1 mM MgCl_2_, and 1 mM ATP). The purified proteins were stored at 4 °C before use.

### Citrullination of proteins

Recombinant PAI-2 protein was citrullinated after incubation with 10 mM CaCl_2_ and rabbit PADI (Sigma-Aldrich) at a concentration of 20 IU per 1 mg protein for 2 h.

### Detection of citrullinated proteins by anti-modified citrulline antibody (anti-MC)

The citrullinated protein was detected by Western blotting using modified anti-citrulline detection kit (Upstate Biotechnology, Lake Placid, NY, USA). Briefly, the citrulline residues of the proteins immobilized on the PVDF sheet after 10% SDS-PAGE were modified by 2,3-butanedione monoxime and antipyrine in a strong acid solution. The modified citrulline residues were visualized using anti-MC in accordance with the manufacturer’s instructions.

### PSMB1 overlay assay

Binding of PSMB1 to naïve form and citrullinated PAI-2 (citPAI-2) were analyzed using PSMB1 overlay assay previously described [18]. Briefly, PAI-2 and citPAI-2 (2.5 μg) were electrophoresed by sodium dodecyl sulfate polyacrylamide gel electrophoresis (SDS-PAGE) and transferred to PVDF membrane. The PVDF membrane was incubated with a buffer containing 10 mM Tris-HCl (pH 7.4), 2% (*w*/*v*) dried milk, 0.1% Tween 20. Filters were washed with phosphate-buffered saline containing 0.2% Nonidet P-40 and then incubated with phosphate-buffered saline/Nonidet P-40 containing 2 μg/mL recombinant PSMB1-GST for 2 h at 4 °C. Filters were washed with phosphate-buffered saline/Nonidet P-40. Bound GST-tagged PSMB1 was detected by anti-GST antibodies using Western blot analysis described previously.

### Preparation of human monocyte-derived macrophages

All participants signed an informed consent under a study protocol approved by the institutional review board of Dalin Tzu Chi Hospital, Buddhist Tzu Chi Medical Foundation (No. B10604013). Heparinized venous blood from healthy individuals was mixed with one-fourth volume of 2% dextran solution (MW 464 kDa; Sigma-Aldrich) and incubated at room temperature for 30 min. Leukocyte-enriched supernatants were collected and layered over a Ficoll-Hypaque density gradient solution (specific gravity 1.077; Pharmacia Biotech, Uppsala, Sweden). After centrifugation, peripheral blood mononuclear cells (PBMCs) were aspirated from the interface. These PBMCs were differentiated to macrophages using Macrophage Generation Media (PromoCell, Heidelberg, Germany) according to the manufacturer’s protocol.

### Statistical analysis

Data were expressed as mean ± standard deviation. Statistical significance was assessed by Student’s *t* test. All statistical analyses were performed using Stata/SE version 8.0 for Windows (StataCorp, College Station, TX, USA). Two-tailed *P* values < 0.05 are considered significant.

## Results

### Characteristic of the differentiated macrophages

**Fig. 1:**
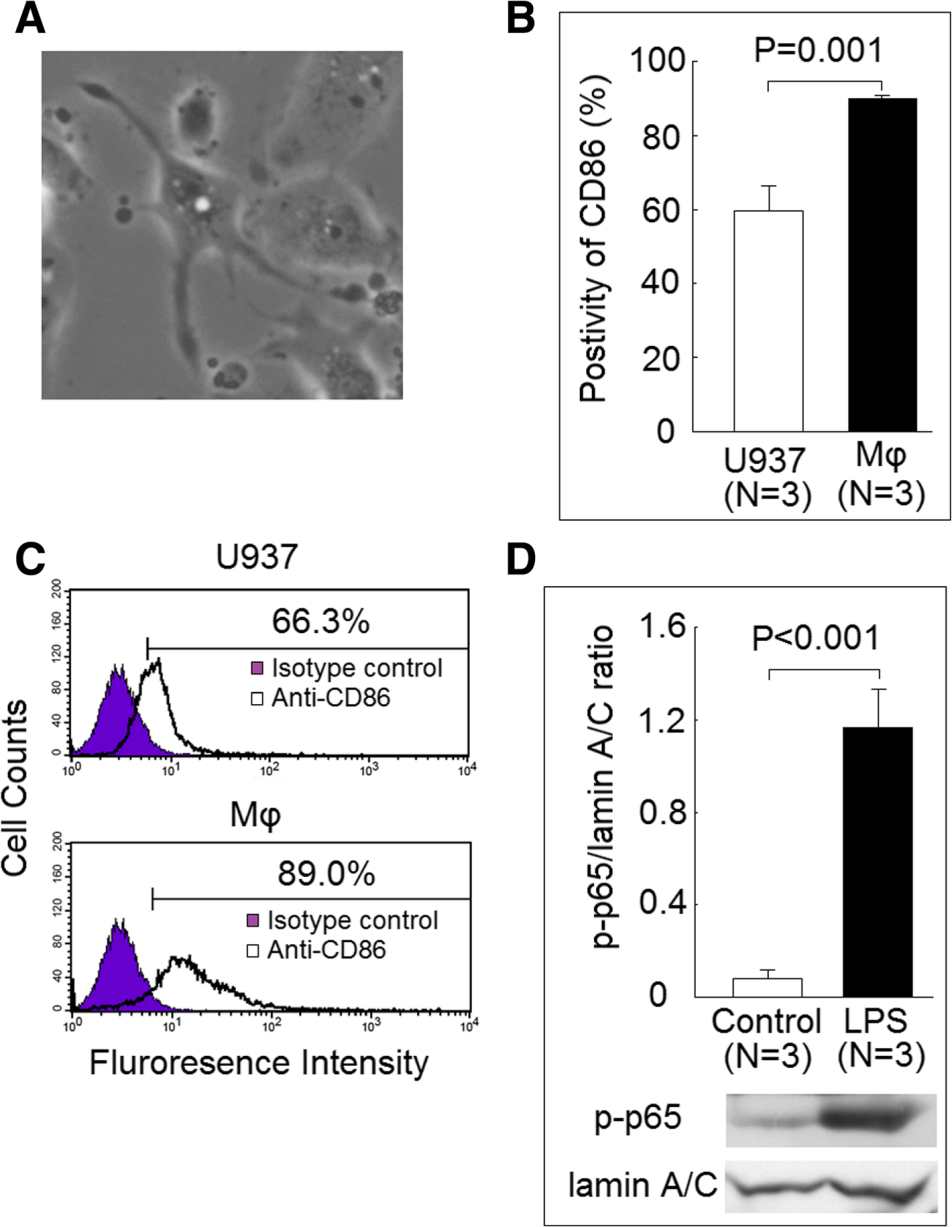
Characteristic of the differentiated macrophages. **a** The rounded, non-adherent U937 cells were transformed to adherent flatten differentiated macrophages with elongated arms stimulated with 500 ng/mL phorbol 12-myristate 13-acetate (PMA) for 48 h. **b** The expression of CD86 was significantly increased in differentiated macrophages compared with U937 cells. **c** A representative example. **d** The addition of LPS could significantly increase the protein levels of phosphorylated p65 subunit of NF-κB (p-p65) in nuclear extract of differentiated macrophages

After cocultured with PMA for 48 h, the rounded, non-adherent U937 cells were transformed to adherent flatten differentiated macrophages with elongated arms (Fig. 1a). The percentage of adherent cells in the differentiated macrophages was 91.3 ± 0.6%. The expression of CD86 was increased in differentiated macrophages compared with U937 cells (Fig. 1b, c). The addition of LPS could markedly increase the protein levels of phosphorylated p65 subunit of NF-κB in nuclear extract (Fig. 1d).

### PADI2 and PADI4 expression and histone 3 citrullination in U937 cells, differentiated macrophages, and macrophages stimulated with LPS

**Fig. 2:**
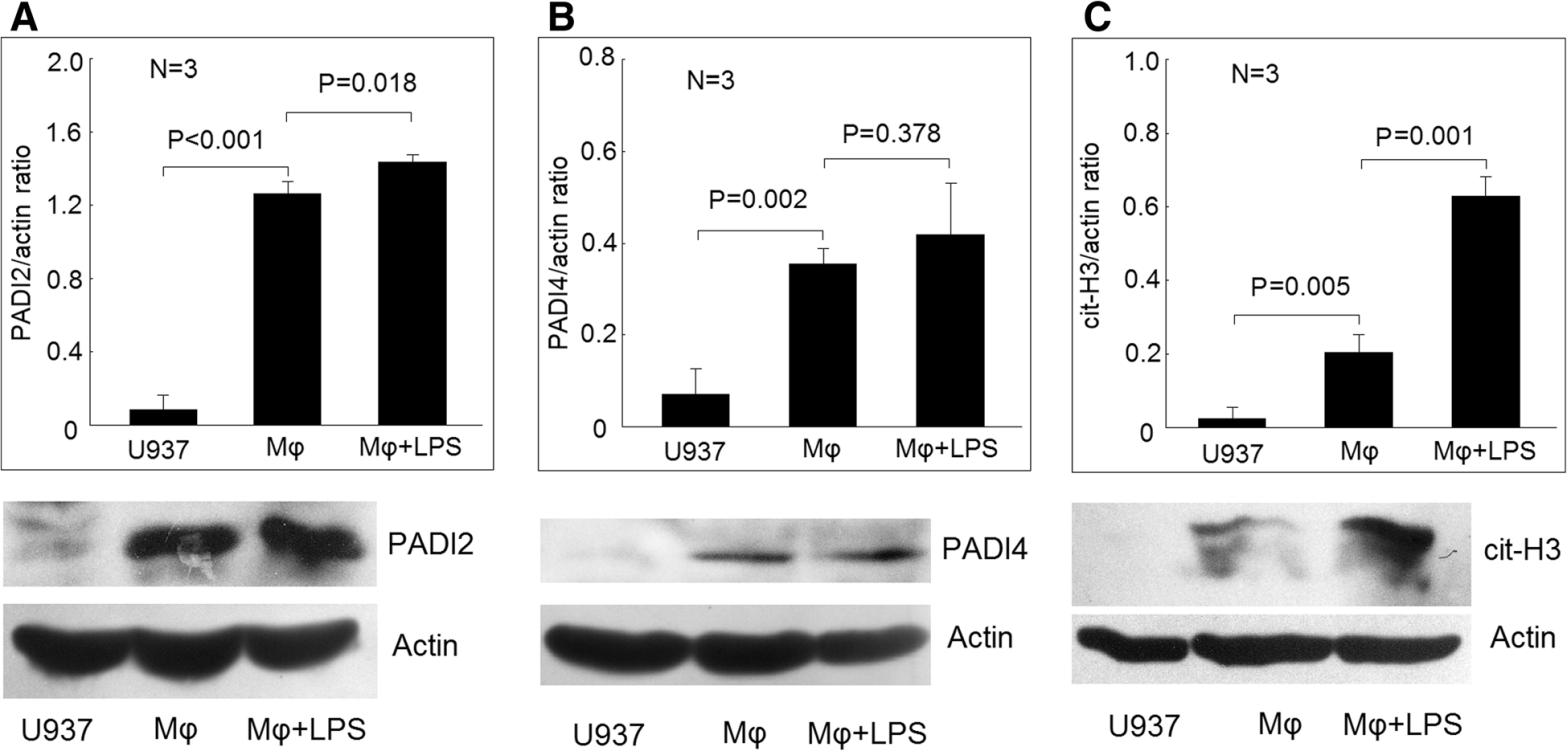
Protein expression of PADI2, PADI4, and citrullinated histone 3 in U937 cells, differentiated macrophages, and macrophages stimulated with lipopolysaccharides (LPS). **a** The protein expression of PADI2 increased in differentiated macrophages compared with U937 cells as detected by Western blot analysis. The addition of LPS slightly increased the protein expression of PADI2 in macrophages. **b** The protein expression of PADI4 increased in differentiated macrophages compared with U937 cells. The addition of LPS did not affect the protein expression of PADI4 in macrophages. **c** The protein level of citrullinated histone 3 (cit-H3) slightly increased in differentiated macrophages compared with U937 cells. The addition of LPS significantly increased the protein level of cit-H3 in macrophages

We found that the protein expression of PADI2 and PADI4 increased markedly in differentiated macrophages compared with U937 cells. The addition of LPS to macrophages modest increased PADI2, but not PADI4 protein expression (Fig. 2a, b). For the evaluation of the effect of PADIs on protein citrullination, we used citrullinated histone 3 as a marker. We found that the protein level of citrullinated histone 3 increased slightly in differentiated macrophages compared with U937 cells. The addition of LPS to macrophages significantly increased the amount of citrullinated histone 3 protein compared with differentiated macrophages cultured with medium only (Fig. 2c).

### Effect of PADI inhibitors on proinflammatory cytokines secretion in differentiated macrophages stimulated with LPS

**Fig. 3:**
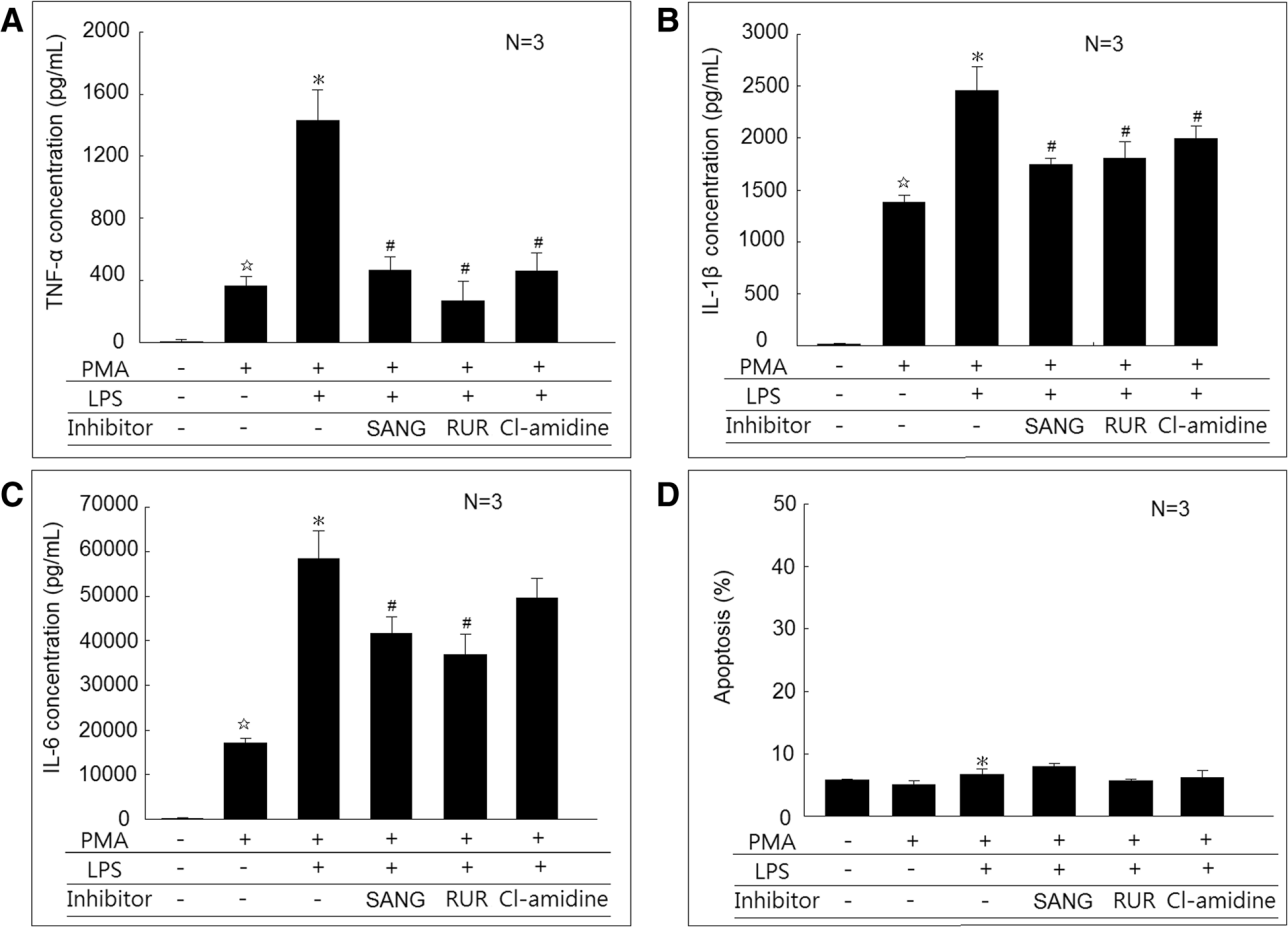
Effects of PADI inhibitors on proinflammatory cytokines secretion in macrophages stimulated with lipopolysaccharides (LPS). **a** U937 cells, differentiated macrophages, and macrophages stimulated with LPS for 24 h, macrophages cocultured with LPS and sanguinarine (SANG), ruthenium red (RUR), or Cl-amidine for 24 h. The culture soups were collected for further analysis of **a** TNF-α, **b** IL-1β, and **c** IL-6 concentration using ELISA. **d** U937 cells, differentiated macrophages, and macrophages stimulated with LPS for 24 h, macrophages cocultured with LPS and SANG, RUR, or Cl-amidine for 48 h. The apoptotic rate of these cells was measured using flow cytometry analysis. ^☆^*P* < 0.05 compared with U937 cells. *P < 0.05 compared with differentiated macrophages. ^#^*P* < 0.05 compared with differentiated macrophage stimulated with LPS

**Fig. 4:**
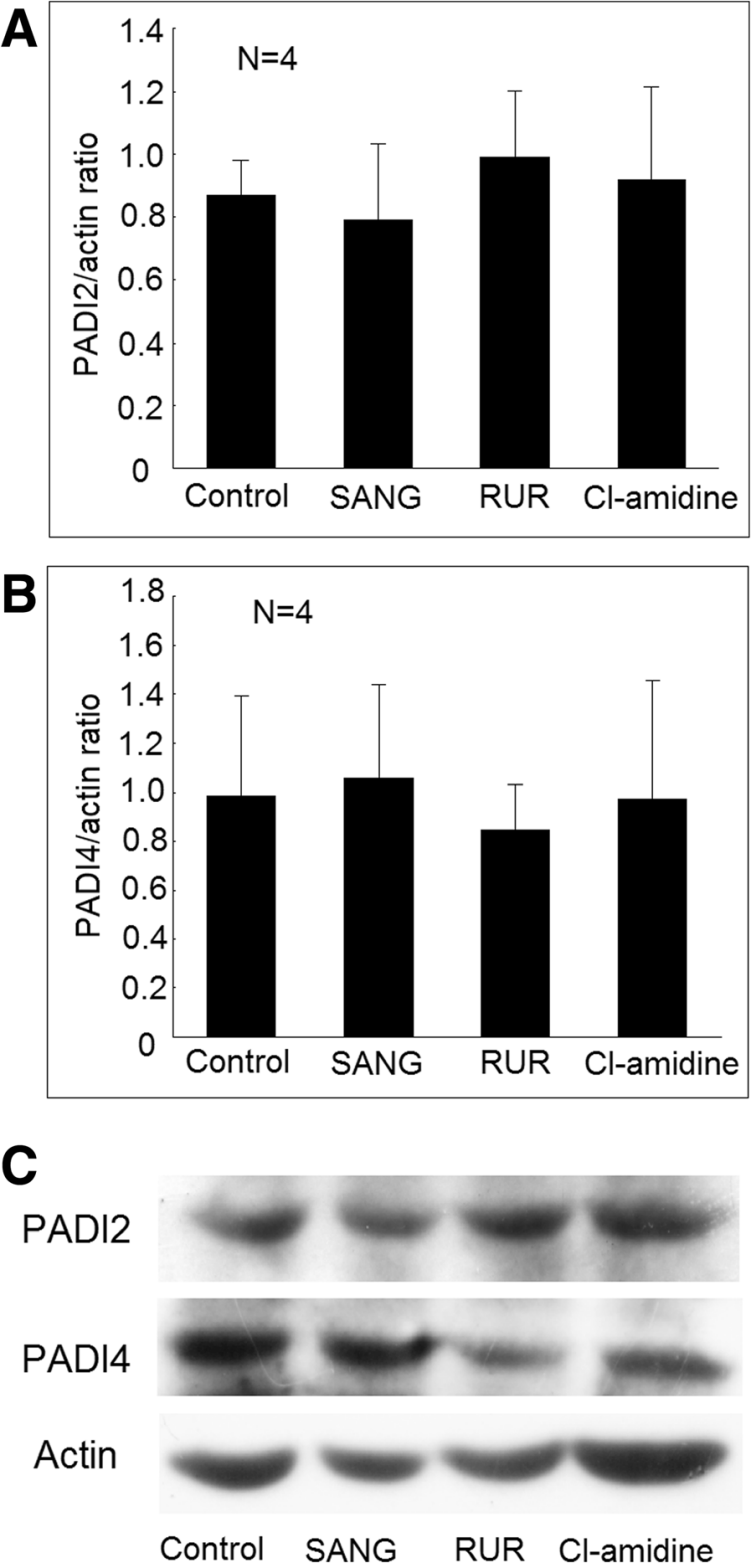
Effects of PADI inhibitors on PADI2 and PADI4 expression in macrophages stimulated with lipopolysaccharides (LPS). **a** The protein expression of PADI2 was not different in LPS-stimulated macrophages cocultured with sanguinarine (SANG), ruthenium red (RUR), or Cl-amidine compared with the control. **b** The protein expression of PADI4 was not different in LPS-stimulated macrophages cocultured with SANG, RUR, or Cl-amidine compared with the control. **c** A representative example

We confirmed that the TNF-α, IL-1β, and IL-6 secretion increased in differentiated macrophages compared with U937 cells. The addition of LPS could significantly increase the secretion of TNF-α, IL-1β, and IL-6 (Fig. 3). The addition of PADI inhibitors, including sanguinarine (SANG), or ruthenium red (RUR) could all significantly suppress TNF-α, IL-1β, and IL-6 cytokines secretion from differentiated macrophages stimulated with LPS (Fig. 3a–c). The addition of Cl-amidine could significantly suppress TNF-α and IL-1β, but not IL-6 cytokines secretion from differentiated macrophages stimulated with LPS. For the viability of the cells, there were no differences between the U937 cells and differentiated macrophages. The addition of LPS to differentiated macrophages could slightly increase the cell apoptosis compared to those cultured without LPS (6.7 ± 0.8% vs. 5.1 ± 0.5%; *P* < 0.05). The addition of SANG, RUR, or Cl-amidine did not affect cell viability compared to macrophages cultured with LPS only (Fig. 3d). Moreover, the addition of SANG, RUR, or Cl-amidine did not affect the expression of PADI2 and PADI4 in macrophages cultured with LPS only (Fig. 4a–c).

### Identification of the citrullinated proteins in U937 cells after being differentiated to macrophages

**Fig. 5:**
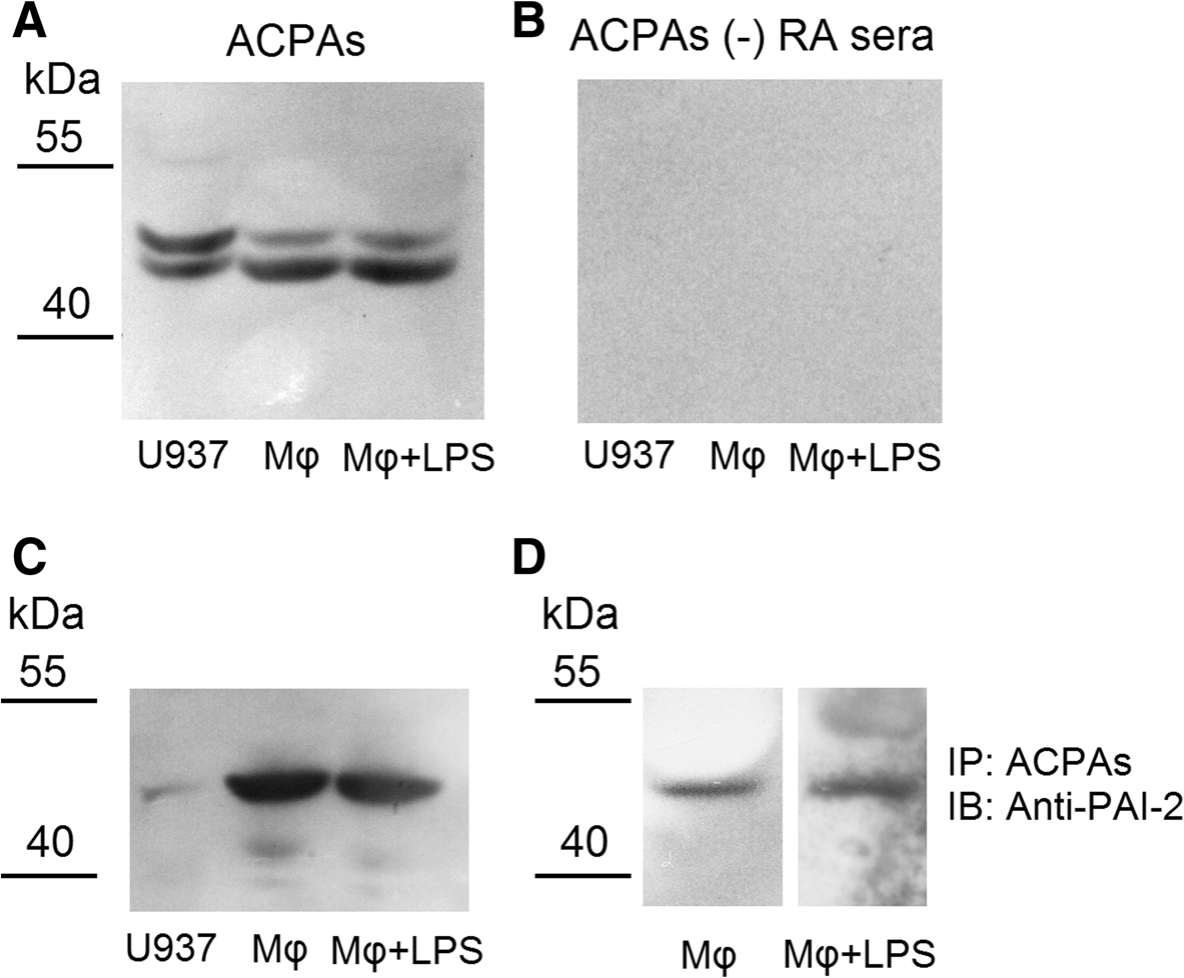
Identification of the citrullinated proteins in U937 cells after being differentiated to macrophages. **a** ACPAs bound to cell protein extract of U937 cells, differentiated macrophages, and macrophages stimulated with LPS for 24 h in 50 kDa and 48 kDa bands. **b** APCAs (−) RA sera did not bind to protein extract of U937 cells, differentiated macrophages, and macrophages stimulated with LPS. **c** Following excision, these bands were sent for proteomic analysis. After series Western blot analysis, we confirmed that antibody against plasminogen activator inhibitor-2 (PAI-2) reacted with cell protein extract of U937 cells, U937 differentiated macrophages, and macrophages stimulated with LPS in the 48 kDa band and the same pattern with those recognized by ACPAs. **d** We used self-purified ACPAs as a probe to immunoprecipitated citrullinated protein from cell lysate of the differentiated macrophages and macrophages stimulated with LPS. We showed the presence of PAI-2 in these citrullinated proteins after Western blot analysis using anti-PAI-2 as a probe

Since PADI2, PADI4, and citrullinated histone 3 were increased in differentiated macrophages compared with U937 cells. We proposed that certain intracellular proteins might be citrullinated during macrophages differentiation and activation. Western blot analysis of U937 cell protein lysate probed by ACPAs revealed at least two distinct protein molecules (molecular weights around 50 kDa and 48 kDa) that consistently reacted with ACPAs compared with ACPA-depleted RA sera (Fig. 5a, b). The 48 kDa band was particularly increased in differentiated macrophages compared with U937 cells. Following excision, these bands were sent for proteomic analysis. The results were plasminogen activator inhibitor-2 (PAI-2), optineurin, zinc phosphodiesterase ELAC protein 2, four and a half LIM domains protein 5, and EF-hand domain-containing protein 1 in the 48 kDa band, and alpha-enolase, zinc finger protein 175, glypican-6, hexokinase-2, synaptic vesicle glycoprotein in the 50 kDa band. We checked the protein expression of these genes, and only the expression of PAI-2 was located at the 48 kDa, and fulfilled the results from previous Western blot (Fig. 5c). We used self-purified ACPAs as a probe to immunoprecipitate citrullinated protein from cell lysate of the differentiated macrophage and macrophages stimulated with LPS. We confirmed the presence of PAI-2 in these citrullinated proteins after Western blot analysis using anti-PAI-2 antibody as a probe (Fig. 5d).

### Using siRNA to inhibit the expression of PAI-2 and its effect on cytokines expression

**Fig. 6:**
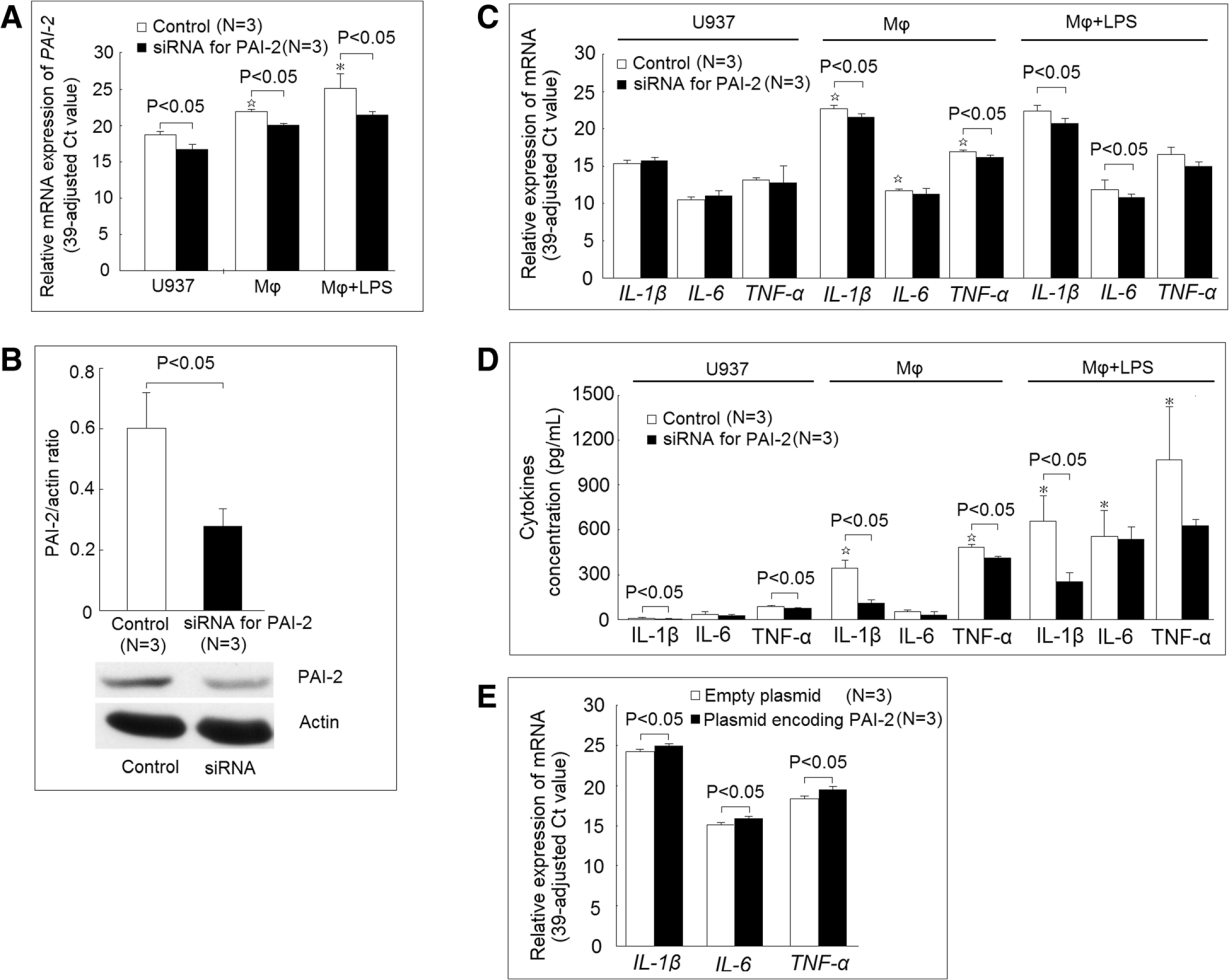
Using siRNA to inhibit the expression of plasminogen activator inhibitor 2 (PAI-2) and its effect on cytokines expression. **a** The transfection of siRNA targeting PAI-2 could effectively suppress the mRNA expression of *PAI-2* in U937 cells, differentiated macrophages, and macrophages stimulated with LPS. **b** The protein expression of PAI-2 was significantly lower in differentiated macrophages after being transfected with siRNA targeting PAI-2. **c** The mRNA expression levels of *IL-1β*, *IL-6*, and *TNF-α* in U937 cells, differentiated macrophages, and macrophages stimulated with LPS transfected with siRNA targeting PAI-2 compared with the controls. **d** The cytokines secretion of IL-1β, IL-6, and TNF-α in U937 cells, differentiated macrophages, and macrophages stimulated with LPS transfected with siRNA targeting PAI-2 compared with the controls. **e** The mRNA expression levels of *IL-1β*, *IL-6,* and *TNF-α* in the macrophage stimulated with LPS after transfected with of siRNA targeting PAI-2 together with plasmid encoding *PAI-2* or empty plasmid. ^☆^*P* < 0.05 compared with U937 cells. *P < 0.05 compared with differentiated macrophages

We found that the mRNA expression of *PAI-2* was increased in differentiated macrophage compared with U937 cells. The addition of LPS further increased the expression of mRNA expression of *PAI-2* in differentiated macrophages (Fig. 6a). We also found that the mRNA expression of *PAI-2* was significantly decreased after transfected siRNA targeting PAI-2 compared with those transfected with scramble oligonucleotides (as a control group) in U937 cells, differentiated macrophages, and differentiated macrophages stimulated with LPS (Fig. 6a). We further confirmed that siRNA targeting PAI-2 significantly suppressed the protein expression of PAI-2 in differentiated macrophages (Fig. 6b). We then measured the mRNA expression of *IL-1β*, *IL-6*, and *TNF-α*. We found that the mRNA expression of *IL-1β*, *IL-6*, and *TNF-α* were all significantly elevated in differentiated macrophages compared with U937 cells, but the addition of LPS did not further increase the mRNA expression of *IL-1β*, *IL-6*, and *TNF-α*. There were no differences between the mRNA expression levels of *IL-1β*, *IL-6*, and *TNF-α* between the U937 cells transfected with siRNA targeting PAI-2 compared with the control group. The mRNA expression levels of *IL-1β* and *TNF-α* but not *IL-6* were significantly lower in the differentiated macrophage transfected with siRNA targeting PAI-2 compared with those transfected with scramble oligonucleotides (as control groups). In the differentiated macrophage stimulated with LPS, the mRNA expression levels of *IL-1β*, *IL-6*, but not *TNF-α* were significantly lower when transfected with siRNA targeting PAI-2 compared with the control group (Fig. 6c). Finally, we found that the cytokine concentrations of IL-1β, IL-6, and TNF-α were all significantly elevated in differentiated macrophages compared with U937 cells. The addition of LPS further increased the cytokine concentrations of IL-1β, IL-6, and TNF-α in differentiated macrophages. Transfection of siRNA targeting PAI-2 suppressed the IL-1β and TNF-α secretion of U937 cells compared with the control group. In the differentiated macrophage, the transfection of siRNA targeting PAI-2 suppressed the IL-1β and TNF-α, but not IL-6 secretion compared with the control group. In the macrophages stimulated with LPS, the transfection of siRNA targeting PAI-2 suppressed the IL-1β, but not TNF-α or IL-6 secretion compared with the control group (Fig. 6d). Then, we transfected siRNA targeting PAI-2 together with plasmid encoding PAI-2 or empty plasmid into macrophages. In macrophages stimulated with LPS, we found the mRNA expression levels of IL-1β, IL-6, and TNF-α were significantly increased in those transfected with siRNA targeting PAI-2 and plasmid encoding PAI-2 compared to those transfected with siRNA targeting PAI-2 and empty plasmid (Fig. 6e).

### Effect of PADI2 expression on PAI-2 and citrullinated PAI-2 (citPAI-2) protein levels

**Fig. 7:**
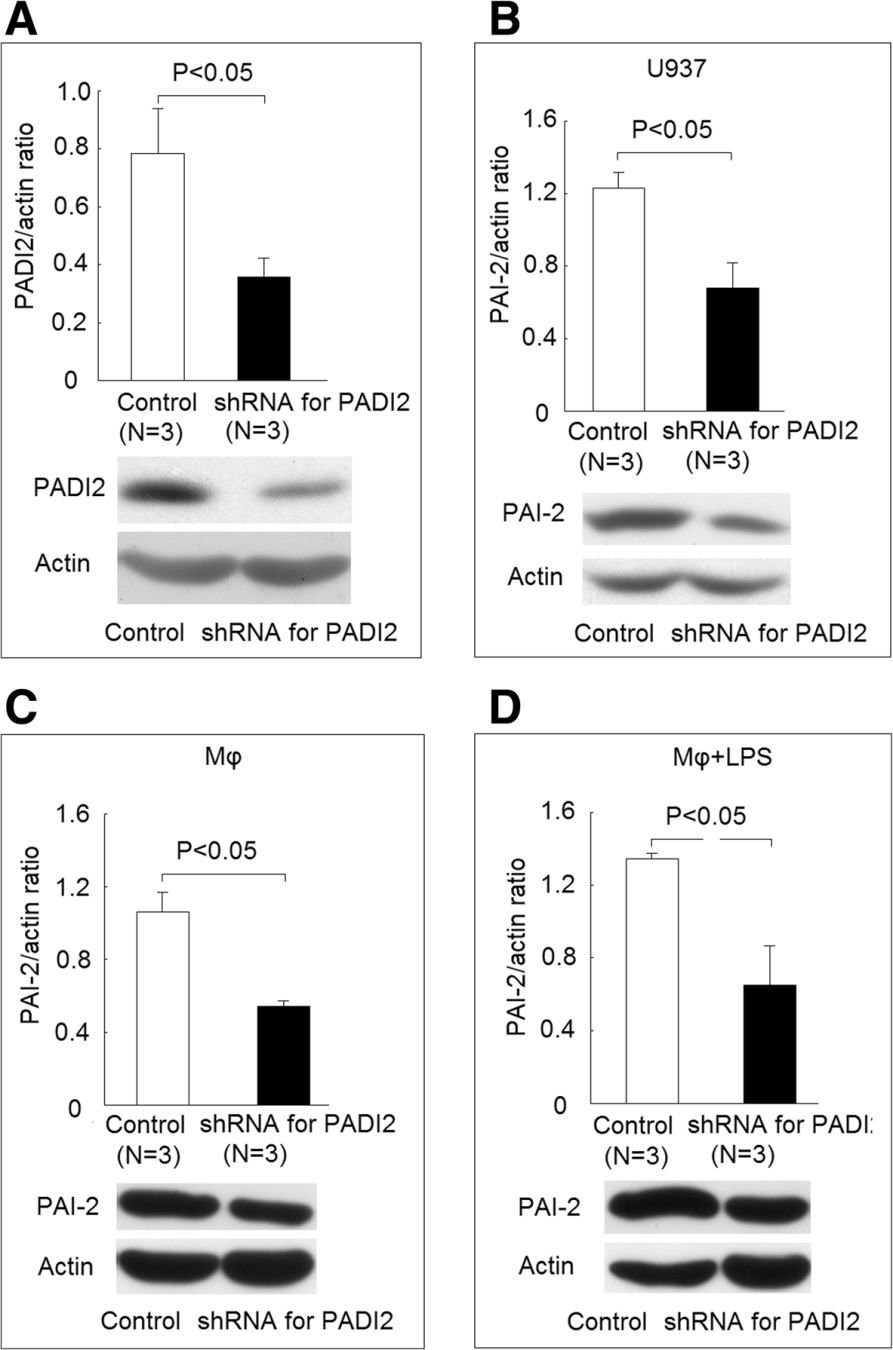
Using short hairpin (sh) RNA to inhibit the expression of PADI2 and its effect on PAI-2 protein expression. **a** Protein expression of PADI2 decreased in U937 cells transfected PADI2 shRNA plasmid compared with those transfected with control plasmid. **b** Protein expression of PAI-2 decreased in U937 cells transfected with PADI2 shRNA plasmid compared with the controls (empty plasmid). **c** Protein expression of PAI-2 decreased in differentiated macrophages transfected with PADI2 shRNA plasmid compared with the controls. **d** Protein expression of PAI-2 decreased in macrophages stimulated with LPS transfected with PADI2 shRNA plasmid compared with the controls

**Fig. 8:**
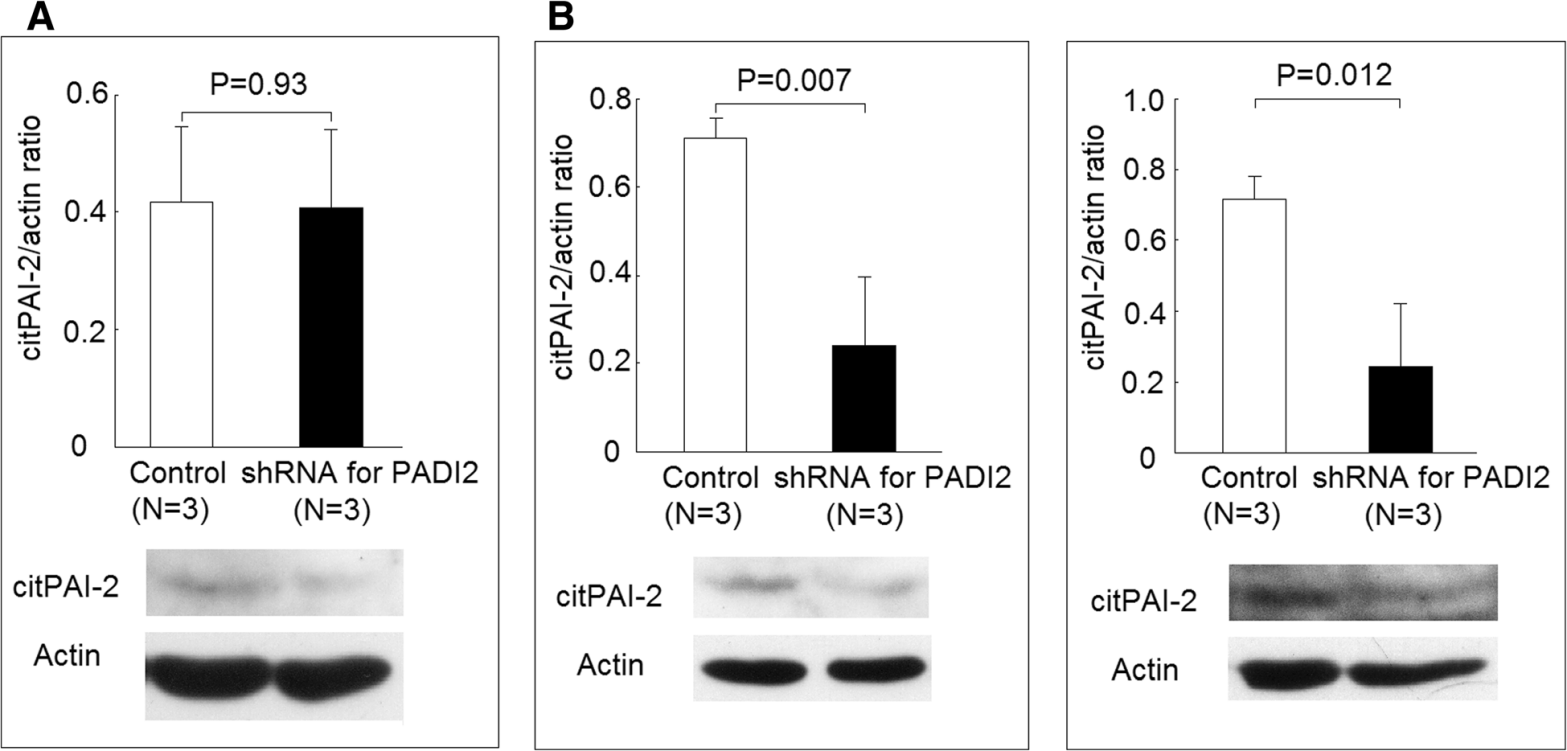
Using short hairpin (sh) RNA to inhibit the expression of PADI2 and its effect on citrullinated PAI-2 (citPAI-2) protein levels. **a** Protein levels citPAI-2 did not change in U937 cells transfected with PADI2 shRNA plasmid compared with the controls (empty plasmid). **b** Protein levels of citPAI-2 decreased in differentiated macrophages transfected with PADI2 shRNA plasmid compared with the controls. **c** Protein levels of citPAI-2 decreased in macrophages stimulated with LPS transfected with PADI2 shRNA plasmid compared with the controls

We found that the protein expression of PADI2 was decreased in U937 cells transfected with PADI2 shRNA plasmid compared with those transfected with control plasmid (Fig. 7a). The decreased expression of PADI2 caused a decreased PAI-2 protein expression in U937 cells (Fig. 7b), differentiated macrophages (Fig. 7c), and differentiated macrophages stimulated with LPS (Fig. 7d). After transfection with PADI2 shRNA plasmid, the protein levels of citPAI-2 did not change in U937 cells due to the low expression of PADIs (Fig. 8a). However, the transfection of PADI2 shRNA plasmid significantly decreased the protein levels of citPAI-2 in differentiated macrophages and macrophages stimulated with LPS (Fig. 8b and c).

### Citrullination of PAI-2 suppressed protein binding PSMB1

**Fig. 9:**
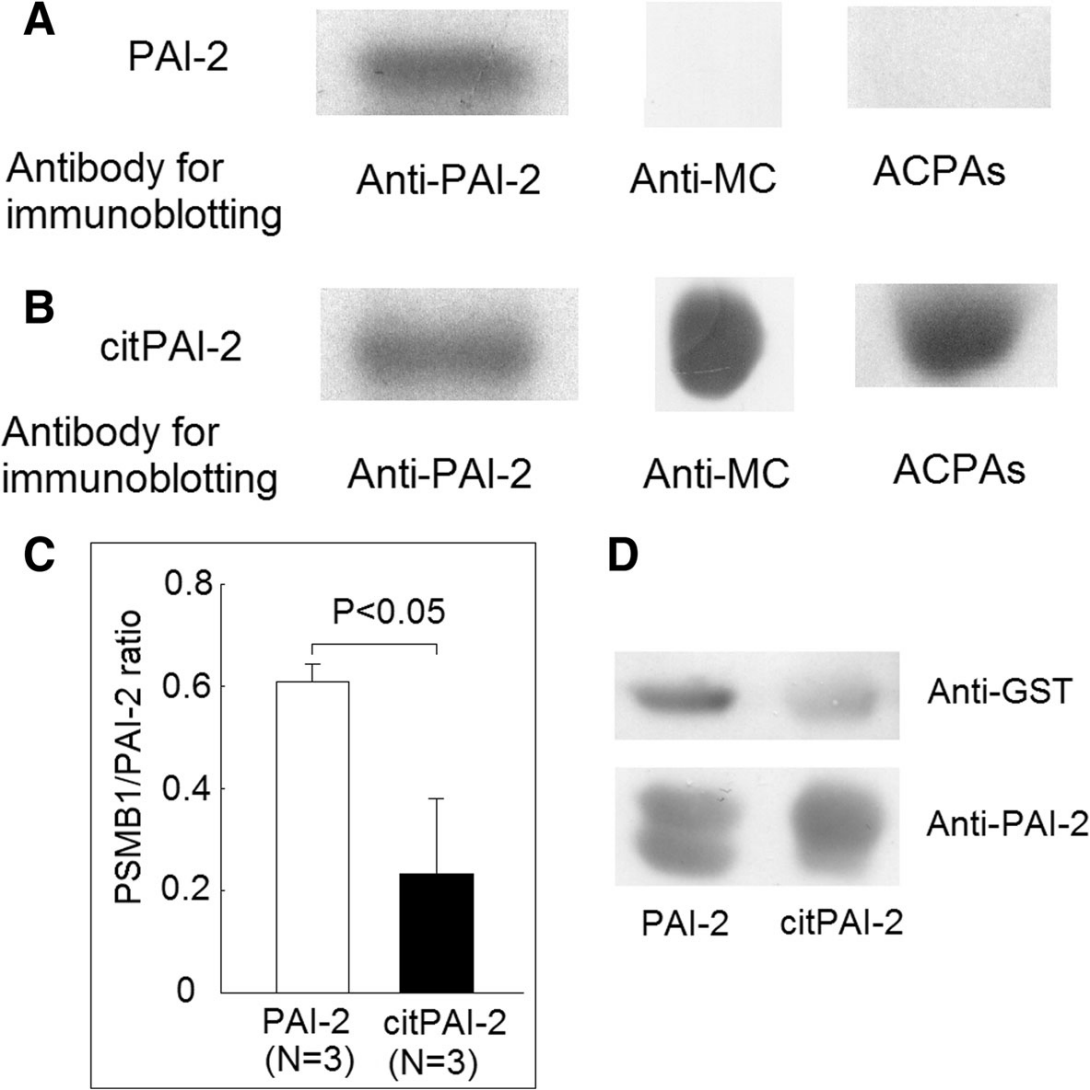
Effects on protein citrullination of PAI-2 in ACPAs and PSMB1 binding ability. **a** Self-cloned PAI-2 was not citrullinated and could not bind to anti-MC and ACPAs. **b** After citrullination, citrullinated PAI-2 could bind to ACPAs and anti-MC. **c** Citrullination of PAI-2 decreased its binding ability to GST tagged PSMB1. **d** A representative example

It is known that PAI-2 could interact with PSMB1 [19] and the binding of PAI-2 with proteasome could affect proteasome function [20]. We investigated whether citrullination of PAI-2 could affect its binding to PSMB1. First, we cloned PAI-2 and GST-tagged PSMB1. We found the self clone PAI-2 was recognized by anti-PAI-2, but not anti-MC or ACPAs (Fig. 9a). After citrullination, citPAI-2 was recognized by anti-PAI-2, anti-MC, and ACPAs (Fig. 9b). We then transferred naïve and citrullinated PAI-2 on the PVDF membrane and incubated with GST-tagged PSMB1. We found that citPAI-2 inhibited its binding ability with PSMB1 (Fig. 9c and d).

### Effect of PADI inhibitors of proinflammatory cytokines secretion in human monocyte-derived macrophages stimulated with LPS

**Fig. 10:**
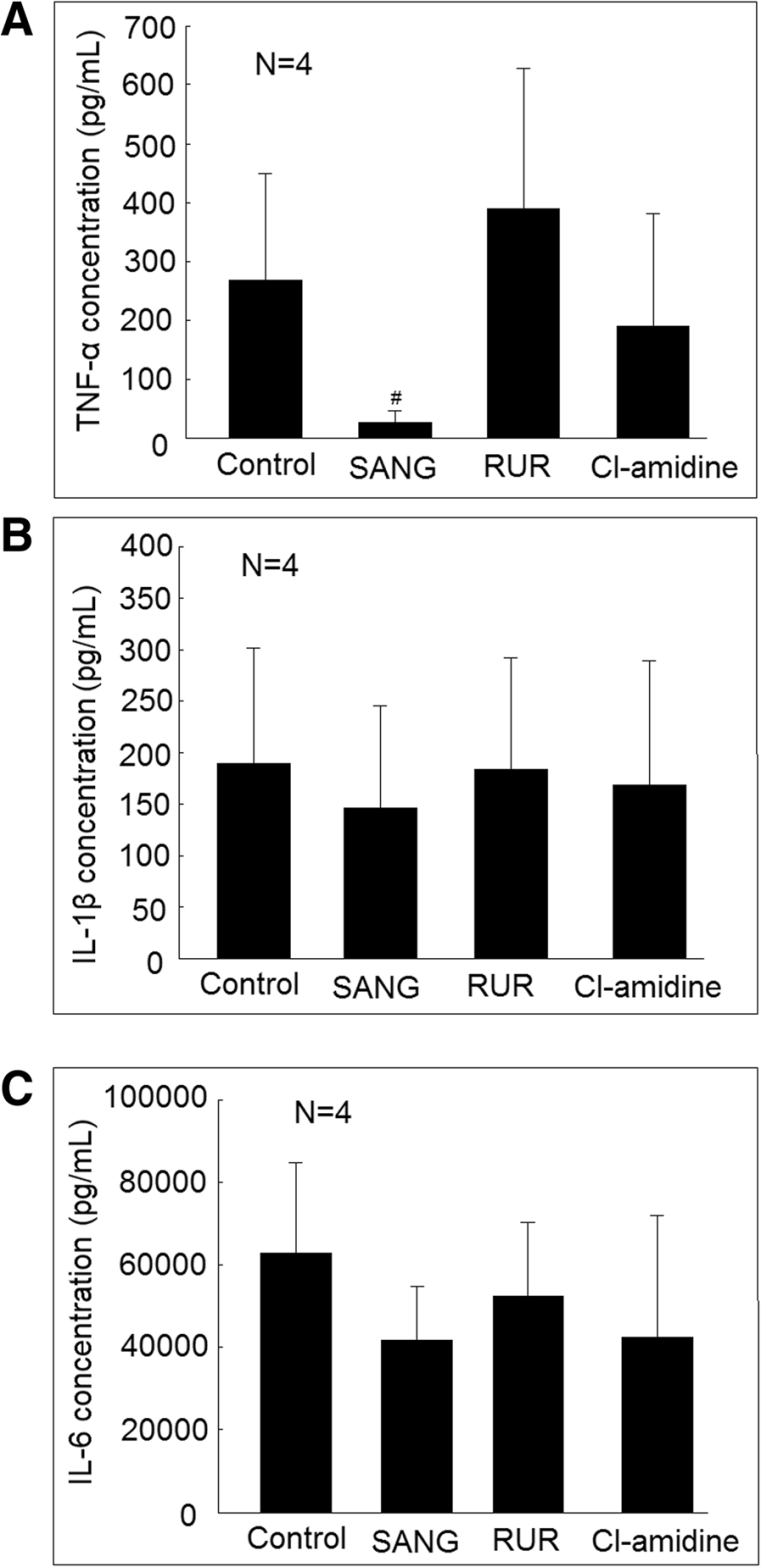
Effects of PADI inhibitors on proinflammatory cytokines secretion in human monocyte-derived macrophages stimulated with lipopolysaccharides (LPS). Human monocyte-derived macrophages cocultured with LPS alone or LPS with 10 μM sanguinarine (SANG), 10 μM ruthenium red (RUR), or 10 μM Cl-amidine for 24 h. The culture soups were collected for further analysis of **a** TNF-α, **b** IL-1β, and **c** IL-6 concentration using ELISA. ^#^*P* < 0.05 compared with human monocyte-derived macrophages stimulated with LPS

**Fig. 11:**
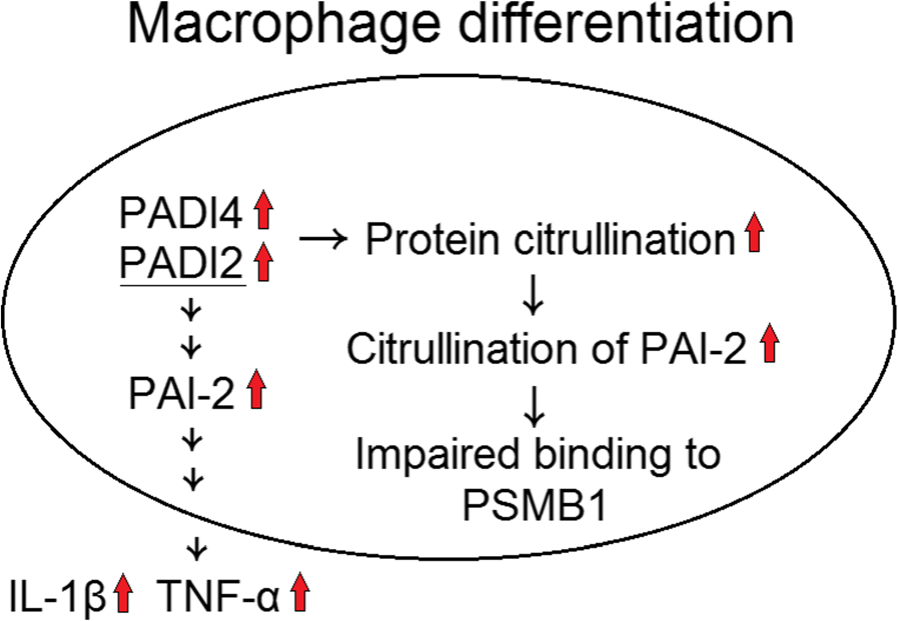
A schematic diagram of this study showing that increased PADI2 and PADI4 expression during the differentiation of macrophages leads to IL-1β and TNF-α production and PAI-2 expression and citrullination. The citrullination of PAI-2 impaired its binding ability with PSMB1

We analyzed the effect of three PADI inhibitors SANG, RUR, or Cl-amidine on proinflammatory cytokine secretion in human monocyte-derived macrophages co-culture with LPS. In a small sample analysis, we found that SANG could significantly suppress LPS-induced TNF-α secretion in human monocyte-derived macrophages (Fig. 10). In conclusion, during macrophage differentiation, the expression of PADI2 and PADI4 increased protein citrullination, including PAI-2 and increased IL-1β and TNF-α production. The citrullination of PAI-2 impaired its binding ability with PSMB1 (Fig. 11).

## Discussion

Our studies showed that the protein expression of PADI2 and PADI4 markedly increased during the monocyte differentiated to macrophage. The increased PADI2 and PADI4 expression citrullinated certain intracellular proteins, including PAI-2. Increased PAI-2 expression, regulated at least by PADI2 promoted the production and secretion of IL-1β and TNF-α. Finally, citrullination of PAI-2 impaired its binding ability to cognate protein, PSMB1.

PADI2 and PADI4 were known to play a critical role in the immunopathogenesis of RA. Inhibition of PADIs is anticipated to be a novel therapeutic target for the treatment of RA [21, 22]. Our study further showed that these three PADI inhibitors could effectively suppress TNF-α secretion, but the effect on the secretion of IL-1β and IL-6 in differentiated macrophages stimulated with LPS was modest and even not significant for Cl-amidine. SANG could effectively suppress LPS-medicated TNF-α secretion of human monocyte-derived macrophages. The clinical application of PADI inhibitors needs further in vivo study. We found that the expression of PADI2 and PADI4 was significantly increased in differentiated macrophages compared with U937 only. The addition of LPS further slightly increased the expression of PADI2, but not PADI4. In contrast, the formation of citrullinated histone 3 was slightly increased in differentiated macrophages compared with the U937 cells. However, the addition of LPS markedly increased the formation of citrullinated histone 3. Therefore, the PADI2 and PADI4 appeared to play a critical role in macrophage differentiation. The stimulation of LPS triggering the Ca^2+^ influx of macrophage could lead to robust formation of citrullinated proteins. We noted that there are some differences compared with a previous study by Vossenaar et al., which showed that the PADI4 expression was similar in freshly isolated monocytes from peripheral blood compared with ex vivo differentiated macrophages of patients with RA and healthy controls [13]. We believe that our differences could be explained by the use of a cell line in our study and a different method of facilitating macrophage differentiation.

We found that there were increased protein citrullination of some 48 kDa proteins and decreased protein citrullination of some 50 kDa proteins during the monocytes differentiated into macrophages (Fig. 5). We only validated one of the 48 kDa proteins, PAI-2, which was indeed citrullinated (Fig. 5), and the citrullination of PAI-2 was necessary for ACPAs binding (Fig. 9). PAI-2, a nonconventional serine protease inhibitor (serpin), is known to greatly increase in monocytes and macrophages after stimulation by LPS [23]. Our study also showed that the protein expression of PAI-2 significantly increased in the differentiated macrophages compared with monocytes (Fig. 5c). It is worth to notice that in Fig. 7, the amount of total proteins needed for performing Western blot analysis of PAI-2 expression in U937 was 50 μg, whereas 10 μg total protein was enough for analysis in macrophages. Recently, numerous serpins, including antiplasmin, antithrombin, tissue plasminogen activator inhibitor, and C1 inhibitor, were identified to be citrullinated in RA synovium and synovial fluid, and these proteins could participate in complement activation and fibrinolysis [24]. The citrullination of these serpins abolishes their ability to perform their function, and our findings also showed that the citrullination of PAI-2 impaired it binding to its cognate protein, PSMB1. As for the biological function of PAI-2, it was initially thought to be an inhibitor of urokinase like PAI-1 [23]. Current studies suggested PAI-2 might have other roles, including host response to viral infection, cell apoptosis, and inflammation [25]. Few studies have addressed the role of PAI-2 in the immunopathogeneis of RA [26, 27]. Our study showed that PAI-2 could promote mRNA expression as well as the secretion of IL-1β and TNF-α, both of which are critical in the immunopathogenesis of RA. Moreover, it is known that PAI-2 could bind to PSMB1 and inhibit proteasome function [19]. Boncela et al. showed that during the inflammatory response, the binding of PAI-2 to PSMB1 would inhibit proteasome function and facilitated cell apoptosis via inhibition of P53 degradation [20]. Our study clearly demonstrated that the citrullination of PAI-2 impaired its binding to PSMB1, which might affect proteasome function during inflammation. Furthermore, we found that decreased PADI2 expression could decrease the expression of PAI-2. Additional studies are needed to further clarify the citrullination on PAI-2 function and the effect of PADIs on the expression of PAI-2.

We noted that in addition to plasminogen activator inhibitor-2, optineurin, zinc phosphodiesterase ELAC protein 2, four and a half LIM domains protein 5, EF-hand domain-containing protein 1, alpha-enolase, zinc finger protein 175, glypican-6, hexokinase-2, and synaptic vesicle glycoprotein were identified from the citrullinated bands in Western blot analysis in monocytes and differentiated macrophages. Among these proteins, alpha-enolase was known to be citrullinated in patients with RA and recognizable by ACPAs [28], but the ACPAs binding ability of other proteins will require further confirmation. We used U397 cells as a model in this study, and therefore, further studies using human monocyte-deviated macrophages are required to clarify our findings.

## Conclusions

Taken together, our findings demonstrated that PADI2 and PADI4 expression increased during macrophage differentiation, and the formation of citrullinated proteins increased in macrophages after stimulation by LPS. PADI inhibitors could suppress LPS-induced proinflammatory cytokine secretion in macrophages. Increased PAI-2 expression during macrophage differentiation promoted inflammatory response and the citrullination of PAI-2 impaired its binding to PSMB1. Thus, our study proposed that PADIs and protein citrullination could play a critical role in the differentiation of macrophage and inflammatory response.

## Abbreviations

ACPAs: Anti-citrullinated protein antibodies
ELISA: Enzyme-linked immunosorbent assay
GSH: Glutathione
GST: Glutathione S-transferase
IL: Interleukin
LPS: Lipopolysaccharides
PADI: Peptidylarginine deiminases
PAI-2: Plasminogen activator inhibitor-2
PMA: Phorbol 12-myristate 13-acetate
PSMB1: Proteasome subunit beta type-1
PVDF: Polyvinyllidene difluoride
RT-PCR: Real-time reverse transcription-polymerase chain reaction
SDS-PAGE: Sodium dodecyl sulfate polyacrylamide gel electrophoresis
Serpin: Serine protease inhibitor
shRNA: Short hairpin RNA
siRNA: Small interfering RNAs
TNF-α: Tumor necrosis factor-alpha

## References

[1] Smolen JS, Aletaha D, McInnes IB (2016). Rheumatoid arthritis. Lancet..

[2] Yu HC, Lu MC (2019). The roles of anti-citrullinated protein antibodies in the immunopathogenesis of rheumatoid arthritis. Tzu Chi Med J.

[3] Rogers GE, Harding HW, Llewellyn-Smith IJ (1977). The origin of citrulline-containing proteins in the hair follicle and the chemical nature of trichohyalin, an intracellular precursor. Biochim Biophys Acta.

[4] Wang S, Wang Y (2013). Peptidylarginine deiminases in citrullination, gene regulation, health and pathogenesis. Biochim Biophys Acta.

[5] Chang HH, Liu GY, Dwivedi N, Sun B, Okamoto Y, Kinslow JD (2016). A molecular signature of preclinical rheumatoid arthritis triggered by dysregulated PTPN22. JCI Insight.

[6] Chang HH, Dwivedi N, Nicholas AP, Ho IC (2015). The W620 polymorphism in PTPN22 disrupts its interaction with peptidylarginine deiminase type 4 and enhances citrullination and NETosis. Arthritis Rheumatol.

[7] Suzuki A, Kochi Y, Shoda H, Seri Y, Fujio K, Sawada T (2016). Decreased severity of experimental autoimmune arthritis in peptidylarginine deiminase type 4 knockout mice. BMC Musculoskelet Disord.

[8] Bawadekar M, Shim D, Johnson CJ, Warner TF, Rebernick R, Damgaard D (2017). Peptidylarginine deiminase 2 is required for tumor necrosis factor alpha-induced citrullination and arthritis, but not neutrophil extracellular trap formation. J Autoimmun.

[9] Iwamoto T, Ikari K, Nakamura T, Kuwahara M, Toyama Y, Tomatsu T (2006). Association between PADI4 and rheumatoid arthritis: a meta-analysis. Rheumatology (Oxford).

[10] Chang X, Xia Y, Pan J, Meng Q, Zhao Y, Yan X (2013). PADI2 is significantly associated with rheumatoid arthritis. PLoS One.

[11] Harre U, Georgess D, Bang H, Bozec A, Axmann R, Ossipova E (2012). Induction of osteoclastogenesis and bone loss by human autoantibodies against citrullinated vimentin. J Clin Invest.

[12] Krishnamurthy A, Joshua V, Haj Hensvold A, Jin T, Sun M, Vivar N (2016). Identification of a novel chemokine-dependent molecular mechanism underlying rheumatoid arthritis-associated autoantibody-mediated bone loss. Ann Rheum Dis.

[13] Vossenaar ER, Radstake TR, van der Heijden A, van Mansum MA, Dieteren C, de Rooij DJ (2004). Expression and activity of citrullinating peptidylarginine deiminase enzymes in monocytes and macrophages. Ann Rheum Dis.

[14] Ferrari-Lacraz S, Sebbag M, Chicheportiche R, Foulquier C, Serre G, Dayer JM (2012). Contact with stimulated T cells up-regulates expression of peptidylarginine deiminase 2 and 4 by human monocytes. Eur Cytokine Netw.

[15] Lu MC, Lai NS, Yin WY, Yu HC, Huang HB, Tung CH (2013). Anti-citrullinated protein antibodies activated ERK1/2 and JNK mitogen-activated protein kinases via binding to surface-expressed citrullinated GRP78 on mononuclear cells. J Clin Immunol.

[16] Lu MC, Lai NS, Chen HC, Yu HC, Huang KY, Tung CH (2013). Decreased microRNA (miR)-145 and increased miR-224 expression in T cells from patients with systemic lupus erythematosus involved in lupus immunopathogenesis. Clin Exp Immunol.

[17] Yu HC, Lai PH, Lai NS, Huang HB, Koo M, Lu MC (2016). Increased serum levels of anti-carbamylated 78-kDa glucose-regulated protein antibody in patients with rheumatoid arthritis. Int J Mol Sci.

[18] Huang HB, Horiuchi A, Watanabe T, Shih SR, Tsay HJ, Li HC (1999). Characterization of the inhibition of protein phosphatase-1 by DARPP-32 and inhibitor-2. J Biol Chem.

[19] Fan J, Zhang YQ, Li P, Hou M, Tan L, Wang X (2004). Interaction of plasminogen activator inhibitor-2 and proteasome subunit, beta type 1. Acta Biochim Biophys Sin Shanghai.

[20] Boncela J, Przygodzka P, Papiewska-Pajak I, Wyroba E, Cierniewski CS (2011). Association of plasminogen activator inhibitor type 2 (PAI-2) with proteasome within endothelial cells activated with inflammatory stimuli. J Biol Chem.

[21] Kawaguchi H, Matsumoto I, Osada A, Kurata I, Ebe H, Tanaka Y, et al. Peptidyl arginine deiminase inhibition suppresses arthritis via decreased protein citrullination in joints and serum with the downregulation of interlukin-6. Mod Rheumatol. 2018. 10.1080/14397595.2018.1532545. 10.1080/14397595.2018.1532545 30285515

[22] Koushik S, Joshi N, Nagaraju S, Mahmood S, Mudeenahally K, Padmavathy R (2017). PAD4: pathophysiology, current therapeutics and future perspective in rheumatoid arthritis. Expert Opin Ther Targets.

[23] Schwartz BS, Bradshaw JD (1992). Regulation of plasminogen activator inhibitor mRNA levels in lipopolysaccharide-stimulated human monocytes. Correlation with production of the protein. J Biol Chem.

[24] Tilvawala R, Nguyen SH, Maurais AJ, Nemmara VV, Nagar M, Salinger AJ (2018). The rheumatoid arthritis-associated citrullinome. Cell Chem Biol.

[25] Medcalf RL, Stasinopoulos SJ (2005). The undecided serpin. The ins and outs of plasminogen activator inhibitor type 2. FEBS J.

[26] Yang C, Huang F (2000). Detection and significance of plasminogen activator inhibitor in synovial tissue, synovial fluid and plasma from patients with rheumatoid arthritis. Chin J Intern Med (Beijing).

[27] Soler Palacios B, Estrada-Capetillo L, Izquierdo E, Criado G, Nieto C, Municio C (2015). Macrophages from the synovium of active rheumatoid arthritis exhibit an activin A-dependent pro-inflammatory profile. J Pathol.

[28] Kinloch A, Lundberg K, Wait R, Wegner N, Lim NH, Zendman AJ (2008). Synovial fluid is a site of citrullination of autoantigens in inflammatory arthritis. Arthritis Rheum.

